# Developing comparative effectiveness studies for a rare, understudied pediatric disease: lessons learned from the CARRA juvenile localized scleroderma consensus treatment plan pilot study

**DOI:** 10.1186/s12969-019-0350-5

**Published:** 2019-07-15

**Authors:** Suzanne C. Li, Robert C. Fuhlbrigge, Ronald M. Laxer, Elena Pope, Maria F. Ibarra, Katie Stewart, Thomas Mason, Mara L. Becker, Sandy Hong, Fatma Dedeoglu, Kathryn S. Torok, C. Egla Rabinovich, Polly J. Ferguson, Marilynn Punaro, Brian M. Feldman, Tracy Andrews, Gloria C. Higgins, For the CARRA Registry Investigators, E. Anderson, K. Francis, I. Goh, J. Jaquith, K. Schollaert-Fitch, C. Smith, J. Weiss, J. Wooton

**Affiliations:** 1 Joseph M. Sanzari Children’s Hospital, Hackensack University Medical Center, Imus PC337, 30 Prospect Ave, Hackensack, NJ 07061 USA; 2 Hackensack Meridian School of Medicine at Seton Hall University, Clifton, NJ USA; 3 University of Colorado- Denver and Children’s Hospital Colorado, Denver, CO USA; 4 University of Toronto and The Hospital for Sick Children, Toronto, Canada; 5 Children’s Mercy, Kansas City, MO USA; 6 Texas Scottish Rite Hospital and UT Southwestern, Dallas, TX USA; 7 Mayo Clinic, Rochester, MN USA; 8 Duke University School of Medicine, Durham, NC USA; 9 University of Iowa Stead Family Children’s Hospital, Iowa City, IA USA; 10 Boston Children’s Hospital, Boston, MA USA; 11 UPMC Children’s Hospital of Pittsburgh, Pittsburgh, PA USA; 12 University of Iowa Carver College of Medicine, Iowa City, IA USA; 13 Rutgers School of Public Health, Rutgers University, Newark, NJ USA; 14 The Ohio State University and Nationwide Children’s Hospital, Columbus, OH USA

**Keywords:** Juvenile localized scleroderma, Comparative effectiveness trial, Study design, Consensus treatment plan, Methotrexate, Corticosteroids, Assessment tools

## Abstract

**Background:**

We designed and initiated a pilot comparative effectiveness study for juvenile localized scleroderma (jLS), for which there is limited evidence on best therapy. We evaluated the process we used, in relation to the specific protocol and to the general task of identifying strategies for implementing studies in rare pediatric diseases.

**Methods:**

This was a prospective, multi-center, observational cohort study of 50 jLS patients initiating treatment, designed and conducted by the jLS group of the Childhood Arthritis and Rheumatology Research Alliance (CARRA) from 2012 to 2015. A series of virtual and physical meetings were held to design the study, standardize clinical assessments, generate and refine disease activity and damage measures, and monitor the study. Patients were initiated on one of three standardized methotrexate-based treatment regimens (consensus treatment plans, CTPs) and monitored for 1 year. An optional bio-banking sub-study was included.

**Results:**

The target enrollment of 50 patients was achieved over 26 months at 10 sites, with patients enrolled into all CTPs. Enrolled patients were typical for jLS. Study eligibility criteria were found to perform well, capturing patients thought appropriate for treatment studies. Minor modifications to the eligibility criteria, primarily to facilitate recruitment for future studies, were discussed with consensus agreement reached on them by the jLS group. There were marked differences in site preferences for specific CTPs, with half the sites treating all their patients with the same CTP. Most patients (88%) completed the study, and 68% participated in the bio-banking substudy.

**Conclusions:**

We demonstrate the feasibility of our approach for conducting comparative effectiveness research in a rare pediatric disease. Multi-center collaboration by dedicated investigators who met regularly was a key factor in the success of this project. Other factors that facilitate these studies include having a sufficient number of investigators to enroll in each regimen, and streamlining study approval and management.

## Background

Pediatric rheumatology encompasses many diseases, which vary greatly in their clinical features, prevalence, and pathophysiology. Identifying effective therapies for patients with rare and complex disorders is challenging, requiring collaborative effort and innovative trial design [1–4]. Juvenile localized scleroderma (jLS) is a rare pediatric rheumatic disease, with an estimated annual incidence of 1–3 per 100,000 children [5, 6]. Within the cross-sectional Childhood Arthritis and Rheumatology Research Alliance (CARRA) Legacy registry of pediatric rheumatology patients in North America, 386 patients with jLS were enrolled between 2010 and 2014 compared to 6607 with juvenile idiopathic arthritis, 1217 with childhood systemic lupus erythematosus, and 688 with juvenile dermatomyositis [7, 8].

Lack of consensus on methods of assessment, logistical difficulties in enrollment, and limited funding are among the factors making randomized clinical treatment trials (RCT) difficult in rare childhood rheumatic diseases. As an alternate way to advance knowledge of best care, CARRA has endorsed and supported comparative effectiveness research studies [3]. This method of prospective observational research allows multiple centers to collectively study patient outcomes during the course of routine patient care, enabling a larger number and wider range of patients to be studied at a lower cost than the typical RCT. In this type of design, statistical analyses that account for potential confounders are key. Comparative effectiveness research also allows optimized regimens of commonly used treatments for rare diseases to be reliably compared to newer medications. With these goals in mind, consensus treatment plans (CTPs) and pilot comparative effectiveness studies have been developed and published by CARRA investigators for several diseases including systemic juvenile idiopathic arthritis and systemic lupus proliferative nephritis [3, 9, 10]. The process we used to develop and implement a pilot comparative effectiveness study in jLS may be useful as a model for implementing such studies in other rare diseases.

Treatment of jLS is particularly difficult to study because of variability in clinical subtypes (including plaque morphea, linear scleroderma, Parry Romberg syndrome, pansclerotic morphea, and mixed forms), lesion severity and location, and the character and severity of extracutaneous manifestations (including joint contractures, hemiatrophy of limbs, facial hemiatrophy, ocular dysfunction, and seizures) [11–13]. Although schemata to measure skin changes in jLS have been developed and validated [14], training is required to establish accuracy and reliability of scoring features such as skin thickening [15].

Treatments for pediatric rheumatic diseases are typically studied in adults before being investigated in children. However, major differences between adult and juvenile onset localized scleroderma, including subtype predominance, frequency and character of extracutaneous manifestations, and typical disease duration indicate that pediatric-specific trials are needed [13]. While there is evidence of shared pathogenesis between LS and systemic sclerosis (SSc) [16, 17], optimal treatment for SSc has also not been determined, and it is far from certain that best treatment for jLS would be the same.

Variations in perceived severity and prognosis can also lead to variations in clinical care, making retrospective analysis of efficacy difficult or impossible. Although there are numerous case series in the literature supporting use of methotrexate (MTX) with or without corticosteroid (CS) for jLS, there is only one multicenter, double-blinded RCT in which methotrexate was shown to be superior to placebo. One significant aspect of this study is that all 70 patients were concurrently treated with oral prednisone for the first 3 months, regardless of treatment arm [18]. A survey of North American pediatric rheumatologists indicated agreement on the use of MTX as the systemic treatment of choice for jLS; however, recommended doses, routes and regimens varied widely with 58 different MTX regimens suggested for the same linear scleroderma of the limb case vignette [19, 20]. Most responders also included an initial course of CS, but again there was a lack of consensus on dose, route, and duration with 59 different corticosteroid regimens suggested for the linear scleroderma case vignette [19, 20]. Clearly, more standardization of even commonly used treatments is necessary for treatment efficacy research in jLS to proceed, and was the motivation for development of this CARRA pilot jLS CTP.

The overall goals of this pilot study were to test the methodology for a larger comparative effectiveness study of jLS within CARRA, and to demonstrate the feasibility of a larger study. The current manuscript describes only part of the CTP pilot study; namely, the planning process, development and post-study evaluation of enrollment criteria, success of enrollment as related to problems with study initiation, the persistence of patients in the study, the three treatment plans, and the standardization of clinical assessment tools for data collection. Subsequent manuscripts will focus on the findings of the study including demographics of participants, performance of the clinical assessments, response to treatments, and quality of life measures.

## Methods

### Aim and study design

The jLS workgroup of CARRA has been working to optimize treatment strategies for juvenile localized scleroderma, with comparative effectiveness studies viewed as a possible means for accomplishing this task. To evaluate the feasibility of conducting comparative effectiveness studies of jLS and other rare pediatric diseases, and identify strategies for implementing such studies, the jLS workgroup conducted a pilot prospective, multi-center, observational cohort study of 50 juvenile LS patients with active disease between 2012 and 2015. The CARRA Legacy Registry was used to house the data. Given the nature of the pilot, the relative rarity of the disease, and the size of the patient population treated at the participating institutions, a sample of 50 patients, enrolled over 12 months, was considered to be adequate to demonstrate feasibility and evaluate the data collection methods and assessment tools created by the study investigators [21, 22]. The data collected by this pilot was intended to allow determination of whether a larger, more comprehensive study could be conducted, to inform the sample size calculation for such a study, and to determine how the study assessments and data collection could be improved. The patient eligibility criteria for this study are detailed in Table 1, with LS subtypes defined by Padua criteria [23].

**Table 1:** Working jLS Study Entry Criteria: Inclusion, Exclusion, and Active Disease criteria

Inclusion Criteria:	
1. Localized scleroderma diagnosed by a Pediatric Rheumatologist or Pediatric Dermatologist according to Padua Preliminary Classification criteria; these criteria exclude eosinophilic fasciitis [23]	
2. Fulfill active disease criteria:	
a. Either at least one item from Active disease criteria Group 1, or two from Group 2	
3. Moderate to high disease severity that warrants systemic therapy in the opinion of the treating physician	
a. Includes all subtypes that involve deeper tissue, extensive skin involvement, and/or extracutaneous involvement	
4. Age < 18 years at onset of disease	
5. Age < 21 years at onset of treatment	
Exclusion criteria:	
1. Treated with systemic corticosteroids in the prior 2 weeks	
2. Treated with methotrexate or mycophenolate mofetil within the prior 3 months^a^	
3. Another defined systemic rheumatic disease (e.g., systemic sclerosis)	
4. Intolerance to study medications	
Working Active Disease criteria (used for Pilot Consensus Treatment Plan study)^a^	
Group 1:	
1. New lesion(s) within the prior 3 months, documented by clinician^a^	
2. Extension of an existing lesion within the prior 3 months, documented by clinician^a^	
a. Lesion extension observed in serial photographs or tracings, or detecting > 30% difference in lesion size (maximum length x width).	
3. Documentation of active or progressive deep tissue involvement^a^	
a. Can be by clinical examination, photographs, MRI, or ultrasound	
4. Erythema of moderate or severe level in lesion or an erythematous lesion border	
a. Erythema scoring level based upon LS Scoring Atlas	
5. Violaceous lesion or border color	
a. Can range from lilac ring to deep violaceous color	
Group 2:	
1. Patient or parent report of new lesion OR extension of existing lesion occurring within the prior 3 months^a^	
a. This criterion ONLY applies for new patients (i.e., first visit to clinician’s office).	
2. Erythema of mild level	
a. Erythema scoring level based upon LS Scoring Atlas	
3. Moderate or severe induration of lesion border^a^	
a. Assessed according to modified Rodnan Skin Scoring (mRSS) levels [15]	
4. Tactile warmth of the lesion	
a. Examiner appreciation of temperature difference based upon comparison to control site (unaffected contralateral site if available).	
5. Worsening hair loss in scalp, eyebrow, or eyelashes; documented by clinician^a^	
6. Elevated creatine kinase level in the absence of other source^a^	
7. Lesion biopsy showing active disease (based upon pathologist report. Typically would be presence of lymphocytes, plasma cells, eosinophils, or other white blood cell)	

Study entry criteria used for the jLS Consensus Treatment Plan (CTP) Pilot study

These criteria were previously developed [20] for the purpose of directing comparative effectiveness studies in jLS and were not intended to qualify or disqualify patients for any specific treatment. The LS Scoring Atlas was generated by the LS workgroup of CARRA and contains photographs of patient lesions demonstrating the different visible scored features; > 80% consensus agreement by workgroup members was required for the photograph to be included in the atlas [20]

^a^denotes criteria that were modified or deleted as a result of this study—see Table 2

### Study meetings

Fifteen investigators (14 pediatric rheumatologists and 1 pediatric dermatologist from 10 CARRA sites in the US and Canada) were involved in the development and conduct of this study. All participated in a series of virtual and physical meetings to complete the study design, develop standardized activity and damage measures, and generate disease assessment tools. An initial face-to-face meeting was held in May 2012, where the case report forms (CRFs) were finalized and a workshop was conducted to standardize clinical assessment among the participating investigators. Dr. Thomas Medsger Jr., an authority in scleroderma, led a training session on skin thickness scoring with adult patient volunteers. A reliability and validity study of the cutaneous activity and damage measures developed for this project was conducted using 13 jLS patient volunteers. A discussion was held at the end of the workshop to address scoring questions.

Monthly teleconference meetings were held throughout the period of the study to enhance site involvement and enrollment, ensure consistency of study procedures, and assess and remedy problems as they arose. The PI (SL) offered two call times in most months, in order to accommodate the schedules of all investigators, and distributed summaries of the discussions to all investigators. Nine additional face-to-face meetings were held before, during and after the study, mostly occurring at the annual meetings of CARRA and/or the American College of Rheumatology, which the majority of study investigators attend on an annual basis. A final face-to-face meeting was held in May 2015 to evaluate the performance of developed criteria and measures, based upon available study data.

### Development of disease assessment tools

To conduct successful comparative effectiveness studies, highly sensitive disease assessment tools are needed to identify potentially small but significant differences in the relative efficacy of treatment regimens under study. Since there is a large variation in the availability of expert assessment using technology-based tools (such as the computerized skin score, infrared thermography, laser Doppler flow, Doppler ultrasound, and magnetic resonance imaging) among centers worldwide (reviewed in [24, 25]), we decided to use a manual assessment tool that could be taught to pediatric rheumatologists and dermatologists. Two previously described LS severity measures, LoSSI and mLoSSI, served as basis for development of the assessments used in this study [14, 26, 27]. Additional variables were added based upon their identification as activity variables in another CARRA jLS group prospective study [28]. All of these studies were discussed by the investigators to define the cutaneous measures for use in this study, with ≥ 80% consensus required for all items.

During the study, the investigators developed a morbidity measure to capture extracutaneous involvement, with the intention that the measure could then be tested on the study data. Part of the rationale for developing this measure was to more accurately capture active disease, as some extracutaneous features such as arthritis and uveitis represent disease activity. Case scenarios with photos derived from actual patients, surveys, and the 1000minds technique (https://www.1000minds.com/) were used to generate scoring weights for this measure, with ≥ 80% consensus required for all items.

To increase standardization of scoring, with parent/patient permission we developed an atlas of photographs (scoring atlas) demonstrating all scoring levels for each visually scored lesion features, including those on different Fitzpatrick skin tone types. At numerous on-line and face-to-face meetings, investigators discussed and came to consensus agreement on which photos to include in the atlas [20]. Face-to-face meetings were used to review modules of the extracutaneous measures developed to confirm consensus agreement, train investigators on use, and to review photos relevant to the scoring variables for inclusion in the scoring atlas.

### Treatment plans

**Fig. 1:**
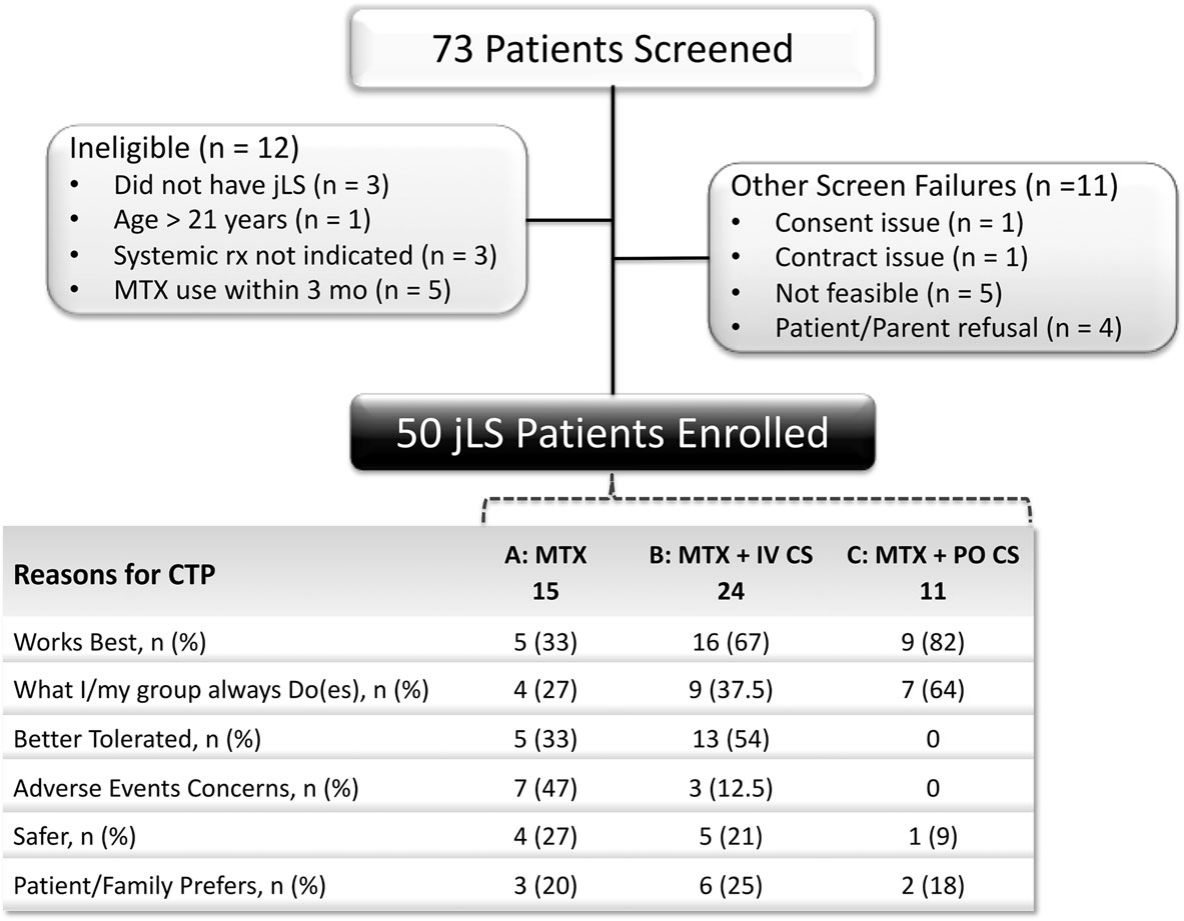
Screen failure and CTP selection reasons. Reasons for screen failures: There was a total of 23 screen failures, with 12 patients not meeting inclusion or meeting exclusion criteria, and 11 not consented for other reasons. “Not feasible” refers to family not consenting because of visit schedule or travel time required to reach to study site. Reasons for choice of CTP: The number of patients treated with each of the CTPs is shown in bold text below the CTP. The physician and patient/family jointly decided upon which CTP to use, and could choose more than one reason for their selection. The reasons for CTP selection are listed, and in the same row the corresponding number (%) of patients enrolled for each reason within each of the three CTPs is shown. Not shown are concerns about patient compliance (*n* = 1, CTP B, 4%), or insurance issues (*n* = 0). CTP: consensus treatment plan; IV CS: intravenous corticosteroid (methylprednisolone); jLS: juvenile localized scleroderma; mo: month; MTX: methotrexate; n: number of patients; rx: treatment; PO CS: oral corticosteroid (prednisone or prednisolone)

We studied 3 methotrexate-based CTPs, designed according to best available evidence, current treatment practices of CARRA members, and consensus methodology [20]. The three CTPs all utilized the same weekly dose of methotrexate (1 mg/kg/weekly dose, maximum 25 mg), with subcutaneous dosing preferred. The CTPs were: methotrexate monotherapy (CTP A), methotrexate with intravenous CS (CTP B, pulse methylprednisolone either once weekly for the first 12 weeks or on three consecutive days per month for the first 3 months, each dose 30 mg/kg with a maximum of 1000 mg/dose); methotrexate with oral CS (CTP C, prednisone or prednisolone, initial 2 mg/kg/day, maximum 60 mg, divided bid for 2–4 weeks, then tapered to 1 mg/kg/day divided bid by 8 weeks, 0.5 mg/kg/day by 16 weeks, 0.25 mg/kg/day by 24 weeks, and off by 48 weeks). There was no plan for tapering or discontinuing methotrexate, as it was intended to be continued throughout the study. Folic or folinic acid supplementation was recommended. The choice of CTP was made jointly by the participating parent/patient and physician (Fig. 1). Since this was an observational study rather than a treatment trial, physicians could change treatment during the study as considered appropriate. Patients were labeled as drop-outs if they withdrew their consent for study participation, did not return for or have study forms completed at follow-up study visits, or self-discontinued study medications before the 9- month visit. Patients were labeled as having a major deviation from the study protocol if they discontinued the study medication for more than 2 weeks, or used a systemic immunomodulator that was not included in the CTP. The latter type of major deviation was considered also to be a treatment failure (the requirement of additional systemic immunomodulator other than specified by their starting CTP).

### Study visits

To avoid inter-rater scoring variability, a given patient was evaluated by the same physician at all study visits. Data collection was performed during regular clinic visits at baseline, and at 2, 4, 6, 9, and 12 months, with a recommended window of +/− 1 month, following initiation of treatment. We planned this large visit window to improve study retention and reduce scheduling difficulties associated with conducting research in busy clinics. Skin activity scoring (modified localized scleroderma severity index [mLoSSI] [14, 29] and localized scleroderma cutaneous activity measure [LSCAM], a measure developed by CTP study group [20]) and physician global assessment of disease activity (PGA-A) were collected at each study visit. Skin damage scoring, PGA-Damage, morbidity scoring, and health related quality of life (HRQoL) measures were captured at 0, 6, and 12 month visits. Collected HRQoL assessments included visual analog or Likert scale questions, PedsQL™ generic, PedsQL™ rheumatology, PedsQL™ Family Impact, childhood dermatology life quality index, and childhood health assessment questionnaire [30–34].

All study participants were entered into the CARRA Legacy Registry, which captured standard information common to pediatric rheumatic diseases, and the majority of scored LS variables. Study data not captured in the Legacy registry were entered into a dedicated electronic database to evaluate the new assessment tools and criteria, and to determine the necessary minimum data set. Using the second database limited the expense associated with use of CARRA Legacy Registry, while enabling capture and testing of all study items.

### Biobanking

For optional biobanking, blood was collected at study entry, 6 months, and 12 months. Specimens were processed for serum, plasma, peripheral blood mononuclear cells, RNA and DNA, and stored at University of Pittsburgh for future study of disease markers. Phlebotomy and shipping supplies were provided by the University of Pittsburgh.

### Regulatory and contract requirements

This study was conducted within the CARRA Legacy registry, with contracting required at each site with the Duke Clinical Research Institute for site payment for data and blood collection. Data identified only by subject number were analyzed at Hackensack University Medical Center, the coordinating center, under ethics approval number for Pro00001481. Individual participating centers obtained their own ethics/institutional review board approvals to enroll patients and collect specimens. Informed consent/assent was obtained per site protocol, and no compensation was given to participants.

### Statistical analysis

Descriptive statistics were used to summarize data on study enrollment and contract completion, study visit times, and evaluate active disease criteria. Detailed patient characteristics, adequacy of sample size, CTP performance, and treatment outcomes will be presented in separate manuscripts.

## Results

### Study enrollment

**Fig. 2:**
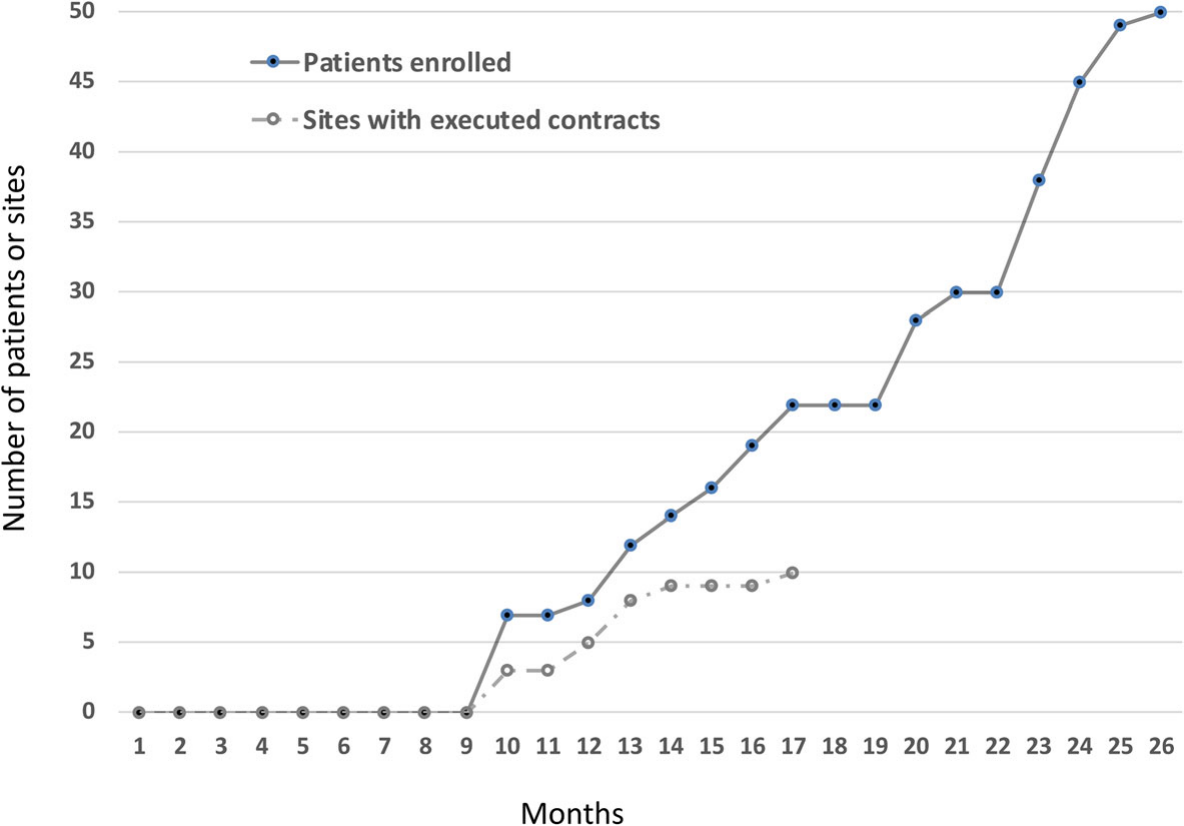
Study enrollment time course: time to obtain site contracts and complete patient enrollment. The black circles show the cumulative number of patients enrolled by month of the study period, with time 0 equal to the start of the grant-funding period. Work on the research study approval and contracts began 4 months before the start of grant funding. The grey open circles show the number of sites with completed study approvals and contracts at each month. The first research study approval and contract completion occurred in the 10th month, with the last occurring at the 17th month

All 15 investigators from the 10 sites enrolled patients (1–9 per site). All patients fulfilled inclusion criteria and did not fulfill any exclusion criteria, as defined in Table 1. IRB and contract approval for the first site occurred at 10 months, and for the final site at the 17th month following study initiation (Fig. 2). Enrollment was completed within 16 months following the first site approval, and within 9 months following the final site approval. Thus, due to administrative delays, full enrollment was not achieved until 26 months from study initiation, and we did not meet our initial goal of full enrollment within 1 year .

Of 73 total patients screened, 23 were not enrolled; 12 because the patient was not eligible (Fig. 1). The most common reason for ineligibility was use of MTX within the past 3 months (*n* = 5). (During this interval, at least two patients were having a disease flare while off MTX but could not be enrolled.) Another 11 patients met inclusion criteria but were not enrolled, most commonly because of study visit schedule and/or travel distance (*n* = 5), or because of patient and/or parent refusal (*n* = 4). One patient/parent refusal was because the family wanted to be treated with a lower dose of MTX than specified in the CTPs; the other 3 were for unstated reasons, with sites queried but unable to furnish additional information. There were no screen failures related to physician preference for a non-CTP treatment regimen.

The general demographics of enrolled patients was similar to published pediatric LS cohorts, with the majority being female (70%), white (92%), and non-Hispanic (82%) [13]. Forty-one of the 50 enrolled patients (82%) had new onset jLS. The remaining 9 had received systemic therapy for jLS in the past and presented with relapse of disease off treatment. Although enrollment of these previously treated patients may have increased the heterogeneity of treatment responses, comparison of efficacy of the different CTPs was not a primary goal.

### Performance of active disease criteria

**Fig. 3:**
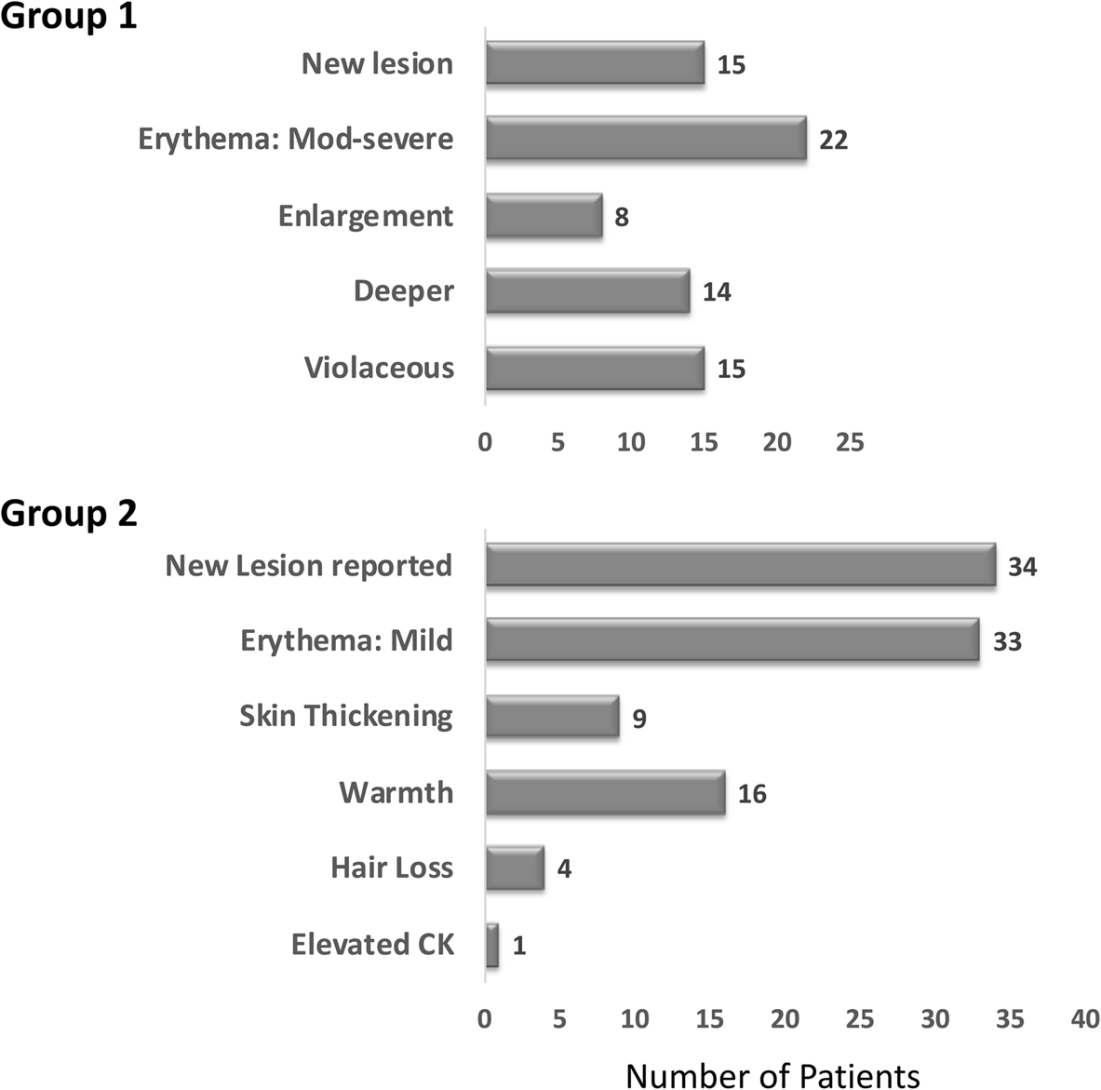
Activity criteria for study inclusion. To be eligible for the study, patients had to fulfill at least 1 criterion from Group 1, or at least 2 criteria from Group 2 (see Table 1). The X axis indicates the number of patients, with the number of patients who fulfilled each criterion shown at the end of each bar. Overall 42 (84%) of patients fulfilled at least 1 of the Group 1 criteria, many of whom also fulfilled Group 2 criteria. Eight (16%) of patients fulfilled only Group 2 criteria. “New lesion” appears in both groups: In Group 1, new lesion must have been documented by physician or physician review of patient photographs. In Group 2, new lesion was not documented by physician, but was reported by patient/parent. “Erythema” appears in both groups: In Group 1, erythema is moderate-severe; In Group 2, erythema is mild

Previously developed active disease criteria for eligibility in a treatment study (Table 1 [20]) were evaluated in this pilot study (Fig. 3). The active disease criteria consist of two categories of features with active disease defined by the presence of a single feature from Group 1, or two or more features from Group 2. All screened patients considered appropriate to treat with systemic immunosuppressants fulfilled at least one active disease criteria group (Group 1 or Group 2, Table 1). Forty-two (84%) of patients fulfilled Group 1 criteria, while 8 (16%) fulfilled only Group 2. The most commonly fulfilled criteria were erythema and new lesion for both groups (Fig. 3). No significant differences were found between patients who fulfilled Group 1 versus those who fulfilled only Group 2 criteria for physician global assessment of disease activity (4 vs. 6, respectively), baseline LS cutaneous activity measure score (6.5 vs. 4), disease duration (15 month vs. 11.5 months), or disease subtype (mostly linear for both groups).

### Initial enrollment into the three CTPs: reasons for choice

**Fig. 4:**
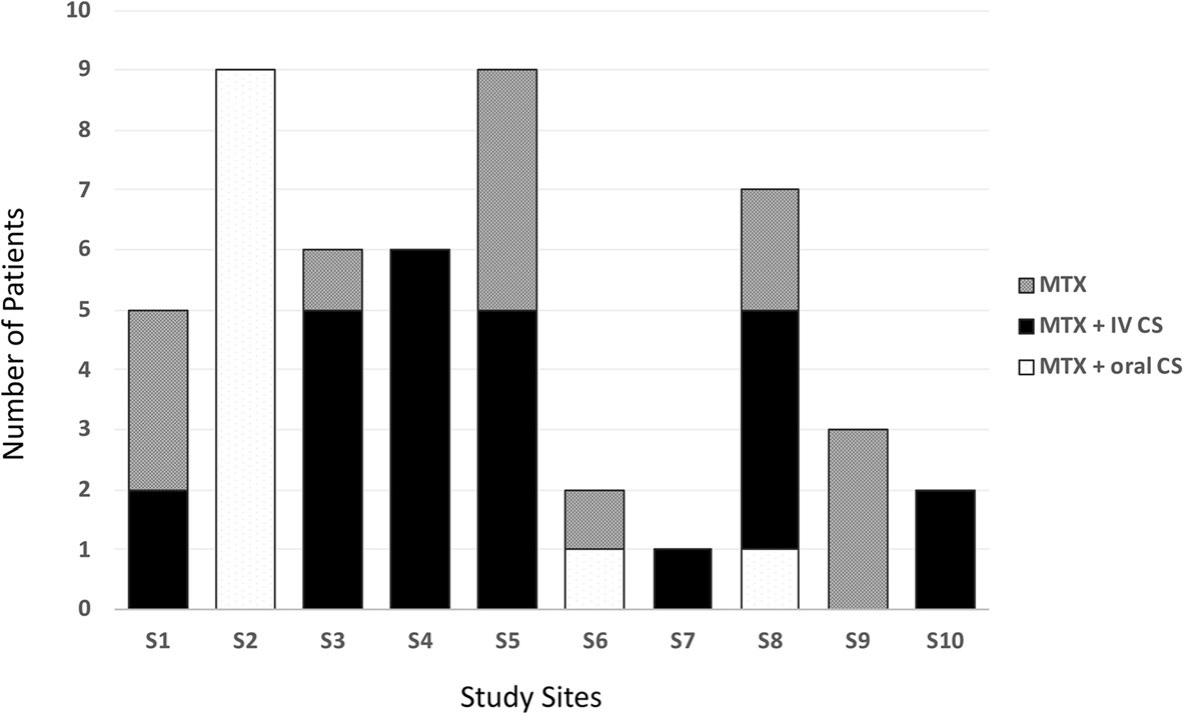
CTP selection by study site. The CTPs selected for the study patients at each study site are shown. Each of the 10 participating CARRA sites is indicated by a different number following the letter “S” (S1-S10). The columns show the number of patients enrolled into each CTP at each site. At 5 sites, all of the study patients at the site were treated with the same CTP, while at four sites two CTPs were used. Only one site (S8) used all three CTPs for treating study patients. CTP: Consensus Treatment Plan; MTX: methotrexate; IV CS: intravenous corticosteroid (methylprednisolone); oral CS: oral corticosteroid (prednisone or prednisolone)

Investigators enrolled patients in all three of the CTPs (Figs. 1 and 4). Not surprisingly, individual site investigators showed strong preferences in choice of CTP (Fig. 4). Nine of 10 centers used only 1 or 2 of the treatment plans available. The most common reason for MTX monotherapy (CTP A) selection was concerns about adverse events, followed by “works best” and “better tolerated.” The most common reason for selection of a corticosteroid and MTX therapy (CTP B or C) was also that it “works best” (Fig. 1).

### Persistence in the study

During the trial, 44 (88%) of the participants remained in the study, while 6 (12%) were considered drop-outs. Reasons for dropping out included the family doubting the need or effectiveness of treatment resulting in withdrawal from the study (*n* = 1) or stopping the medication before the 9 month study visit (*n* = 1), data not being collected after the baseline visit due to not being recognized as a study patient at a satellite clinic [*n* = 1], patient switching care to a non-study physician, either an adult rheumatologist or pediatric rheumatologist located closer to the family (*n* = 2), and unknown (*n* = 1, site queried but unable to furnish reason for drop-out.).

Most participants completed either 5 or 6 of the six scheduled visits (74%). The proportion completing 4, 3, 2, or 1 visit were 10, 4, 2, and 6% respectively. Seventy-nine percent of visits were completed within the 1 month of target window. Completion of the study was defined as the patient remaining on prescribed treatment and completing at least the 9-month visit. Based on this definition, 88% of patients completed the study.

### Post-study evaluation of eligibility criteria

A more critical evaluation of the performance of the inclusion and exclusion criteria (Table 1) was conducted near the end of the study to determine if there should be any modifications for their use in future studies. All modifications for future studies required agreement by ≥ 80% of the investigators. Based on this review, elevated CK was eliminated as an active disease criterion because it was rarely selected and assignment of etiology was considered potentially problematic. Several criteria for active disease were combined to make a single criterion for disease extension to simplify use of the criteria (Table 2, italics, compare to Table 1). The presence of a waxy white or yellow lesion was added as a criterion (Table 2) because it was identified as an active feature in another prospective study [28], but since data analysis for that study was not finished until the after this CTP pilot was already underway, we chose not to add waxy white or yellow as an activity criterion during the course of the CTP. We have tested its association with active disease in the CTP data set, which will be described in a subsequent manuscript.

**Table 2:** Revised jLS Study Entry Criteria: Inclusion, Exclusion, and Active Disease criteria

Inclusion Criteria:	
1. Localized scleroderma diagnosed by a Pediatric Rheumatologist or Pediatric Dermatologist according to Padua Preliminary Classification criteria; these criteria exclude eosinophilic fasciitis [23]	
2. Fulfill active disease criteria:	
a. Either at least one item from Active disease criteria Group 1 or two from Group 2	
3. Moderate to severe disease severity that warrants systemic therapy in the opinion of the treating physician	
a. Includes all subtypes that involve deeper tissue(s), extensive skin involvement, and/or extracutaneous involvement	
4. Age < 18 years at onset of disease	
5. Age < 21 years at onset of treatment	
Exclusion criteria:	
1. Treated with systemic corticosteroids in the prior 2 weeks	
2. Treated with methotrexate or mycophenolate mofetil within the prior *4 weeks*	
3. Another defined systemic rheumatic disease (e.g., systemic sclerosis)	
4. Intolerance to study medications	
Revised Active Disease criteria	
Group 1:	
Developing comparative effectiveness studies	
1. *New, larger, or deeper lesion that developed within the past 3 months associated with erythema, violaceous color, and/or skin thickening. Disease extension must be documented by one of the following*	
a. *Clinical exam*	
b. *Measurements or tracings*	
c. *Photographs (may be provided by family)*	
*d. Imaging: MRI, ultrasound, CT, 3D imager*	
2. Erythema of moderate or severe level	
a. Erythema scoring level based upon LS scoring atlas	
3. Violaceous color	
a. Can range from lilac ring to deep violaceous color	
Group 2:	
1. Erythema of mild level	
a. Erythema scoring level based upon LS scoring atlas	
*2. Waxy white or yellow lesion*	
a. *These lesions have white or yellowish appearance with smooth, waxy feeling*	
b. *They are associated with skin thickening (induration)*	
*3. Skin thickening of lesion*	
a. Assessed according to modified Rodnan Skin Scoring (mRSS) levels [15]	
4. Tactile warmth of the lesion	
a. Examiner appreciation of temperature difference based upon comparison to control site (unaffected contralateral site if available).	
*5. Worsening hair loss on scalp or face*	
6. Inflammation within lesion identified on tissue biopsy	

Revised entry criteria for jLS treatment studies. Modifications of the criteria used for the jLS Consensus Treatment Plan (CTP) Pilot study shown in Table 1; modified criteria are shown in italics. These criteria are not intended to qualify or disqualify patients for any specific treatment. The LS Scoring Atlas was generated by the LS workgroup of CARRA and contains photographs of patient lesions demonstrating the different visible scored features; ≥ 80% consensus agreement by workgroup members was required for the photograph to be included in the atlas [20]

In addition, the group decided to shorten the required wash-out time after treatment with MTX or mycophenolate from 3 months to 1 month for entry into future studies. The impetus behind this decision was the observation that at least 2 patients followed by the investigators experienced recurrence of active disease 1–2 months after stopping MTX but could not be enrolled in the study. Pharmacokinetic studies have shown that MTX polyglutamates in red blood cells have elimination half-lives of ranging from 1.2–4.3 weeks, with median times for MTX polyglutamates to become undetectable ranging from 4 to 10 wks [35]. Thus, it appeared likely that some patients may flare quickly following discontinuation of MTX. Based on the known pharmacokinetics of MMF, showing an even shorter average elimination half-life for mycophenolic acid (8.5 h) and its glucuronide 13 h) [36], we opted to use 1 month as our minimum washout time for MMF as well. The revised eligibility criteria recommended for future jLS treatment studies are shown in Table 2, with italics indicating changes as compared to Table 1.

### Biobanking

Thirty-six (72%) participants/parents consented to biobanking. All three requested samples were collected for 21 patients, 2 samples for 7 patients, and 1 sample for 8 patients. Reasons for failure to complete collection of biobanking samples included blood collection being done at non-affiliated facilities per patient/family preference, lack of facilities at satellite clinics to collect and process samples for mailing, lack of study coordinators to attend to specimen collection and processing, and inability to draw labs at Friday clinics because the biobanking site could not receive samples on a Saturday.

## Discussion

We report here the successful development and enrollment of a pilot comparative effectiveness study for jLS, a rare pediatric rheumatic disease for which there is no proven best treatment regimen. In addition to assessing feasibility, the purpose of this pilot study was to identify how subsequent studies could be improved, and to evaluate new assessment tools. We reached our enrollment goal of 50 jLS patients, and found that the demographics of our study participants were typical for this disease. However, total enrollment time was 26 months, or more than a year longer than expected, with contract issues being the major reason for delay.

The high degree of agreement on the use of these CARRA CTPs for treating jLS patients is demonstrated by there being no screen failures related to physician preference for a non-CTP regimen for treatment. The limited number of screen failures due to refusal demonstrates that patients and parents are willing to participate in comparative effectiveness research, and the low drop-out rate (12%) indicates that these CTPs were generally tolerable to patients and families. As with most clinical studies, the visit and travel schedule precluded several patients from participating, and led some participants to miss study visits. Nonetheless, a relatively high proportion of patients, 44 of 50 (88%), completed the study.

Bio-banked specimens aligned with detailed clinical information are valuable resources for future research in disease pathogenesis and treatment, even more so in rare diseases because they are so difficult to acquire. We demonstrated that bio-banking is feasible in clinic-based comparative effectiveness studies, though several centers were unable to collect or ship specimens at all of the assigned visits, for reasons as given in results. Of 36 patients who contributed to the optional biobanking sub-study, only 21 (58%) provided all three samples per protocol. It will be important in future studies to work on reducing the logistic barriers to sample collection. Nonetheless, the samples collected in this pilot are of significant value to this community of investigators.

One aspect of comparative effectiveness research that reduces barriers to enrollment is that investigators and study patients can choose among standardized treatment options. We noted that sites showed distinct preferences in their choice of CTP, with half the sites treating all of their study patients with the same CTP, and only one site using each of the three CTPs. Part of this lack of variation in intra-center treatment choice may be due to the small numbers enrolled by some centers. In the absence of definitive efficacy studies to provide guidance, physicians may tend to base treatment decisions on their own observational history and/or what they have been taught, rather than on evidence of efficacy. In addition, the rarity of jLS means that many physicians have limited experience with treating jLS. Given the lack of data regarding comparative efficacy, the observation that the reasons for selection of any of the three CTPs were “It works best” or “It is what I/my group always does,” highlight the need for better data.

Our previously developed inclusion and exclusion criteria, including activity criteria for eligibility in a treatment study, seemed to perform well. However, post-study review and consensus agreement led to some modifications of these criteria to potentially increase eligibility of patients to enroll in future treatment studies, and to simplify their use (compare Table 2 italicized text to Table 1).

## Conclusions

We were able to design and fully enroll a pilot prospective, multicenter, 1-year observational cohort study of three standardized methotrexate-based treatment regimens (CTPs) for juvenile localized scleroderma, an uncommon and understudied childhood disease. This pilot study allowed us to evaluate the performance of the study inclusion and exclusion criteria, to propose modifications of those criteria, and to consider protocol changes likely to improve future treatment studies of jLS.

Specifically, “lessons learned” that will help with design of further comparative effectiveness research appeared to fall within the following four general categories:

1. Investigator commitment and effective communication: A committed, cohesive, and enthusiastic group of investigators who are willing to re-think pre-existing assumptions about treatments and methods of evaluation, and who participate in frequent, detailed, well-planned meetings to discuss the protocol, are most likely to be successful in designing and completing difficult studies.
2. Number and variability of participating investigators/sites. Despite the lack of definitive information, investigators showed strong preferences in their treatment choices. Therefore, it is important to have a sufficient number of investigators with different treatment preferences to adequately enroll in all treatment arms of a comparative effectiveness study. It may be advisable to survey investigators as to their treatment preferences in advance to assess if additional investigators/sites are needed to ensure a balanced distribution of patients across the treatment arms.
3. Support of professional organizations. Encouragement, logistical and financial support, and infrastructure from a larger organization were extremely valuable for many aspects of this study, including resources for collection and analysis of data, and facilitating multiple face-to-face meetings of the investigators. Other valuable support for this study included standardization of biospecimen collection and processing protocols to ensure consistency of samples, and optimize their value for future translational studies. As pediatric rheumatology is a rare subspecialty, most pediatric rheumatology centers have few providers and limited staff support, so that backing and support from larger professional organizations is important for the success of clinic-based studies.
4. Institutional administrative challenges. We achieved our target enrollment of 50 jLS patients, but enrollment took more than a year longer than expected due to contract issues with participating medical institutions. Efficient and rational review processes by individual or shared IRBs/Ethics Committees and institutional contracts offices are essential for timely enrollment in medical research studies.

Our description of the elements that supported completion of this study, and some of the problems that we encountered in the course of the study, may be of benefit to other clinicians wishing to develop comparative effectiveness research studies for other rare diseases.

## Data Availability

The datasets generated and/or analyzed during the current study are available from the CARRA Legacy registry on application of a reasonable request.

## Abbreviations

bid: Two times per day dosing
CARRA: Childhood Arthritis and Rheumatology Research Alliance
CK: Creatine kinase
CS: Corticosteroid
CTP: Consensus treatment plan
HRQoL: Health related quality of life
jLS: Juvenile localized scleroderma
kg: Kilograms
LoSSI: Localized scleroderma skin severity index
LS: Localized scleroderma
LSCAM: Localized scleroderma cutaneous activity measure
mg: Milligrams
mLoSSI: Modified localized scleroderma skin severity index
MMF: Mycophenolate mofetil
MTX: Methotrexate
PedsQL™: Pediatric Quality of Life™
PGA-A: Physician global assessment of disease activity
PGA-D: Physician global assessment of disease damage
PO: Oral
RCT: Randomized clinical trial
Rx: Treatment
SSc: Systemic sclerosis
USA: United States of America
